# Auraptene Acts as an Anti-Inflammatory Agent in the Mouse Brain

**DOI:** 10.3390/molecules201119691

**Published:** 2015-11-10

**Authors:** Satoshi Okuyama, Mayu Morita, Miki Kaji, Yoshiaki Amakura, Morio Yoshimura, Koji Shimamoto, Yu Ookido, Mitsunari Nakajima, Yoshiko Furukawa

**Affiliations:** 1 Department of Pharmaceutical Pharmacology, College of Pharmaceutical Sciences, Matsuyama University, 4-2 Bunkyo-cho, Matsuyama, Ehime 790-8578, Japan; sokuyama@cc.matsuyama-u.ac.jp (S.O.); lmv_mayu_laulea@yahoo.co.jp (M.M.); miblue_x102@yahoo.co.jp (M.K.); 16080430m.u@gmail.com (K.S.); kobukurosuki0314@gmail.com (Y.O.); mnakajim@cc.matsuyama-u.ac.jp (M.N.); 2 Department of Pharmacognosy, College of Pharmaceutical Sciences, Matsuyama University, 4-2 Bunkyo-cho, Matsuyama, Ehime 790-8578, Japan; amakura@cc.matsuyama-u.ac.jp (Y.A.); myoshimu@cc.matsuyama-u.ac.jp (M.Y.)

**Keywords:** auraptene, cerebral ischemia, hippocampus, anti-inflammation, astrocytes, cyclooxygenase-2, COX-2

## Abstract

The anti-inflammatory activity of auraptene (AUR), a citrus coumarin, in peripheral tissues is well-known, and we previously demonstrated that AUR exerts anti-inflammatory effects in the ischemic brain; the treatment of mice with AUR for eight days immediately after ischemic surgery suppressed demise and neuronal cell death in the hippocampus, possibly through its anti-inflammatory effects in the brain. We suggested that these effects were at least partly mediated by the suppression of inflammatory mediators derived from astrocytes. The present study showed that (1) AUR, as a pretreatment for five days before and another three days after ischemic surgery, suppressed microglial activation, cyclooxygenase (COX)-2 expression in astrocytes, and COX-2 mRNA expression in the hippocampus; (2) AUR suppressed the lipopolysaccharide-induced expression of COX-2 mRNA and the mRNA of pro-inflammatory cytokines in cultured astrocytes; (3) AUR was still detectable in the brain 60 min after its intraperitoneal administration. These results support our previous suggestion that AUR directly exerts anti-inflammatory effects on the brain.

## 1. Introduction

Auraptene (AUR; 7-geranyloxycoumarin), a simple coumarin contained in the peels of citrus fruits such as grapefruit (*Citrus paradise*), has been reported to have anti-tumor promoting effects [1,2]. In the 21st century, inflammation has been identified as the root cause of various tumors [3,4]. Therefore, the effects of AUR in peripheral macrophages have attracted the attention of researchers [5,6,7]. We recently reported that AUR exerts anti-inflammatory effects not only in peripheral tissues, but also in the brain because it (1) suppresses inflammatory responses in the ischemic brain [8] and (2) ameliorates lipopolysaccharide (LPS)-induced inflammation in the mouse brain [9].

In our previous study [8], we demonstrated using immunohistochemical methods that AUR acts as a neuroprotective agent in the ischemic brain, and its effects may be mediated by the suppression of inflammatory responses. We examined the effects of AUR following the initiation of its administration on the day of ischemic surgery and thereafter for eight days until the day of sacrifice. This time point (brain tissues were prepared on the eighth day after ischemic surgery) was adequate to investigate the neuroprotective effects of AUR, but too late to determine its impact on the transcriptional levels of any inflammatory factors such as inflammatory cytokines and cyclooxygenase (COX)-2. Thus, the first objective of the present study was to evaluate the effects of AUR on brain tissues under the conditions of an earlier time point; *i.e.*, the administration of AUR was initiated five days before ischemic surgery and continued for eight days in the present study (brain tissues were prepared on the third day after ischemic surgery).

We previously demonstrated that COX-2 was expressed in astrocytes in ischemic brains [8] and brains with LPS-induced inflammation [9]. Thus, the second objective of the present study was to determine whether AUR had any anti-inflammatory effects in cultured astrocytes. We treated these cells with LPS, a bacterial endotoxin and generally accepted inducer of pro-inflammatory cytokines [10], in order to induce inflammation.

In our previous studies [8,9], we did not obtain any information to demonstrate that AUR penetrates the brain. Therefore, the final objective of the present study was to establish whether (1) AUR passes through the blood-brain barrier (BBB) and directly exerts anti-inflammatory effects in the brain; or (2) AUR remains in peripheral tissues and indirectly exerts anti-inflammatory effects in the brain.

## 2. Results and Discussion

### 2.1. Suppressive Effects of AUR on Inflammation in the Ischemic Brain

**Figure 1:**
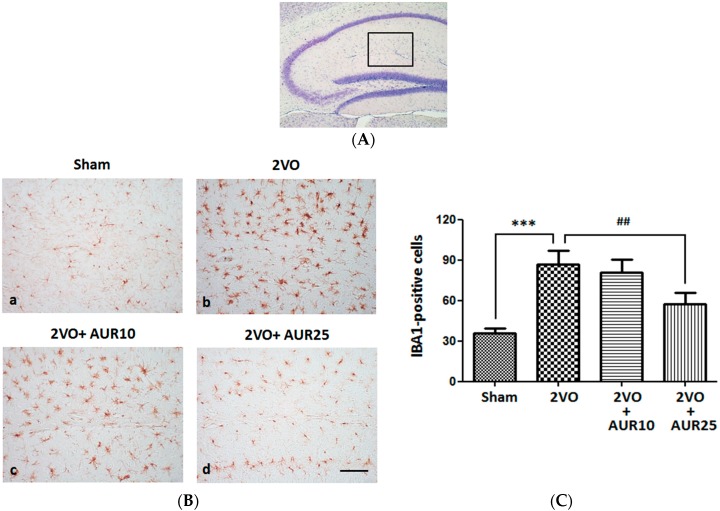
Effects of AUR on the activation of microglia in the hippocampus. (**A**) Region within the hippocampal region for observations of IBA-1-positive cells; (**B**) Representative photographs of IBA-1-positive cells in the indicated groups. The scale bar indicates 100 µm; (**C**) Number of IBA-1-positive cells in the four groups. Values are the means ± SEM (15~18 sections in each group). *** *p* < 0.001; and ^##^ *p* < 0.01, as indicated by the brackets.

Microglial activation generally occurs as an early response in the ischemic brain [11]. Since the expression of ionized calcium binding adaptor molecule 1 (IBA1) was previously reported to be associated with microglial activation in the ischemic brain, its immunostaining is considered useful for evaluating the pathophysiological roles of activated microglia in ischemic injury [12]. Therefore, we subcutaneously (*s.c.*) injected AUR (10 or 25 mg/kg/day) into the mouse for five days before bilateral common carotid artery occlusion (2-vessel occlusion: 2VO) and then for another three days after surgery. Microglial cells in the hippocampus (the stratum lacunosum-moleculare of Ammon’s horn and molecular layer of the dentate gyrus (DG), as shown in Figure 1A) were then stained with the antibody against IBA1. Figure 1B shows that IBA1-positive microglial cells were in a ramified form (an inactive form) in the Sham group, and in a hypertrophied form (an activated form called ameboid microglia) and in greater numbers in the 2VO group. The number of IBA1-positive cells was significantly higher in the 2VO group than in the Sham group (Figure 1C, *** *p* < 0.001). The ramified form of microglia was also observed in the 2VO + AUR10 group (Figure 1B), but at lower numbers in the 2VO + AUR25 group (Figure 1B). The number of IBA1-positive cells in the 2VO + AUR25 group was significantly (^##^ *p* < 0.01) lower than that in the 2VO group (Figure 1C). These results indicated that the treatment with AUR (25 mg/kg/day) for five days before and for another three days after ischemic surgery successfully suppressed microglial activation in the ischemic brain.

**Figure 2:**
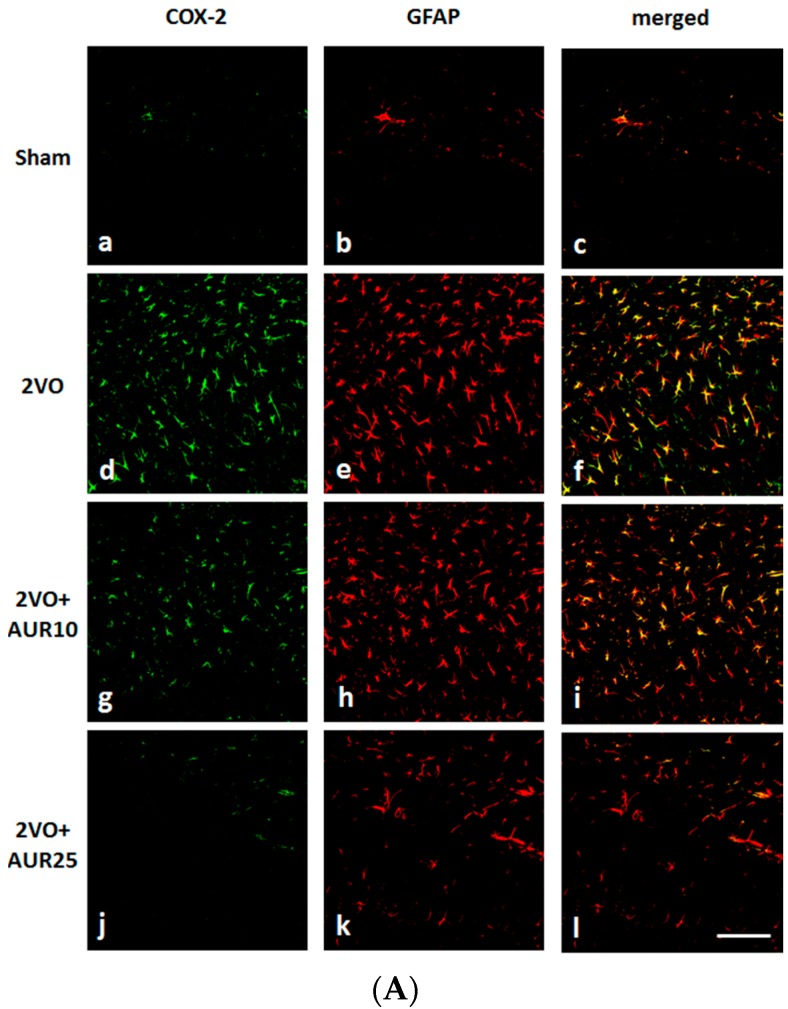

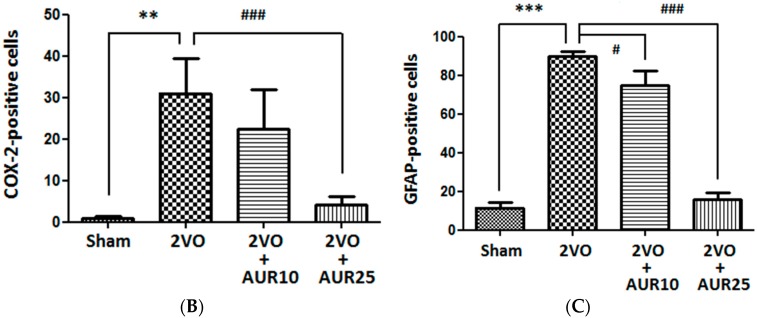
Effects of AUR on the expression of COX-2 and GFAP in the hippocampus. (**A**) Representative photographs of COX-2-positive cells (green; a, d, g, j), GFAP-positive cells (red; b, e, h, k), and cells co-expressing COX-2 and GFAP (yellow; c, f, i, l). The scale bar indicates 100 µm; (**B**) The numbers of COX-2-positive cells; and (**C**) GFAP-positive cells are shown. Values are the means ± SEM (6 sections in each group). ** *p* < 0.01; *** *p* < 0.001; ^#^ *p* < 0.05; and ^###^ *p* < 0.001, as indicated by the brackets.

COX-2 has been implicated in ischemic stroke injury [13]. Figure 2A shows that COX-2 immunoreactivity in the hippocampus (the stratum lacunosum-moleculare of Ammon’s horn and molecular layer of the DG, as shown in Figure 1A) was weak in the Sham group (a), but was strong in the 2VO group (d). The intensity of this immunoreactivity was slightly lower in the 2VO + AUR10 group (g) than in the Sham group (a), and was markedly lower in the 2VO + AUR25 group (j). Figure 2B shows that the number of COX-2-positive cells was significantly higher in the 2VO group (** *p* < 0.01) than in the Sham group, and that the high-dose treatment with AUR (2VO + AUR25) led to significant reductions (^###^ *p* < 0.001) in this number in the 2VO group. These results indicated that the treatment with AUR (25 mg/kg/day) for five days before and for another three days after ischemic surgery suppressed the expression of COX-2.

Figure 2A shows that the expression of glial fibrillary acidic protein (GFAP; a marker of activated astrocytes) was markedly stronger in the 2VO group (e) than in the Sham group (b). GFAP expression levels were lower in the 2VO + AUR10 group (h) than in the 2VO group (e), and were markedly lower in the 2VO + AUR25 group (k). Figure 2C shows differences in the number of GFAP-positive cells between the Sham group and 2VO group, between the 2VO group and 2VO + AUR10 group, and between the 2VO group and 2VO + AUR25 group, all of which were significant (*** *p* < 0.001, ^#^ *p* < 0.05, and ^###^ *p* < 0.001, respectively). In all groups, COX-2-positive cells were immunopositive for GFAP (*viz.* astrocytes) (Figure 2A–C, f, i, and l), but not for IBA-1 (*viz.* microglia) (data not shown). These results indicated that activated astrocytes contributed to the expression of COX-2 three days after ischemic surgery, as described previously [14].

**Figure 3:**
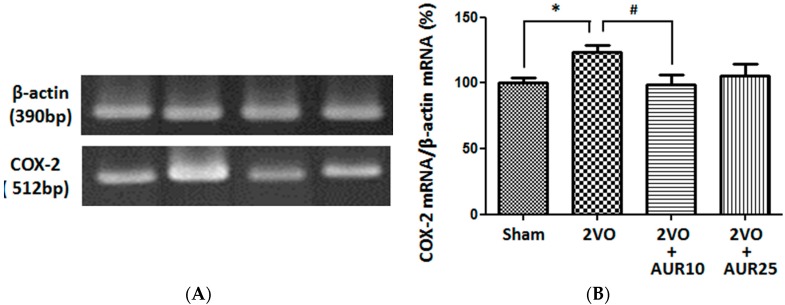
Effects of AUR on the expression of COX-2 mRNA in the hippocampus. (**A**) Densitometric patterns of COX-2 mRNA and β-actin mRNA bands; (**B**) Densitometric quantification of COX-2 mRNA band densities normalized by the density of the β-actin mRNA band. Values are the means ± SEM (5~6 for each group). * *p* < 0.05 and ^#^ *p* < 0.05, as indicated by the brackets.

We then evaluated the effects of ischemia on COX-2 mRNA levels in hippocampal tissues collected three days after 2VO surgery. As shown in Figure 3, 2VO surgery significantly increased COX-2 mRNA levels (* *p* < 0.05) over those observed in the Sham group. The AUR treatment at a lower dose significantly suppressed this increase (^#^ *p* < 0.05), while the higher dose slightly inhibited it. These results suggest that AUR affected not only the translation process, but also the transcription process of COX-2. In macrophages, AUR has been suggested to disturb the translation process, but not the transcription process of COX-2 [7]. The reason for the difference between the reactivities of macrophages and astrocytes currently remains unknown. The mRNA levels of pro-inflammatory cytokines such as interleukin (IL)-1β mRNA and tumor necrosis factor (TNF)-α mRNA were very low, and thus, we were unable to determine whether AUR influences these factors.

CA1 hippocampal neurons were previously reported to be lost several days after transient global ischemia, whereas nearby DG neurons were relatively resistant [8,15,16,17]. Our previous findings indicated that the treatment of ischemic mice with AUR for eight days successfully suppressed demise and neuronal cell death in the hippocampus [8].

**Figure 4:**
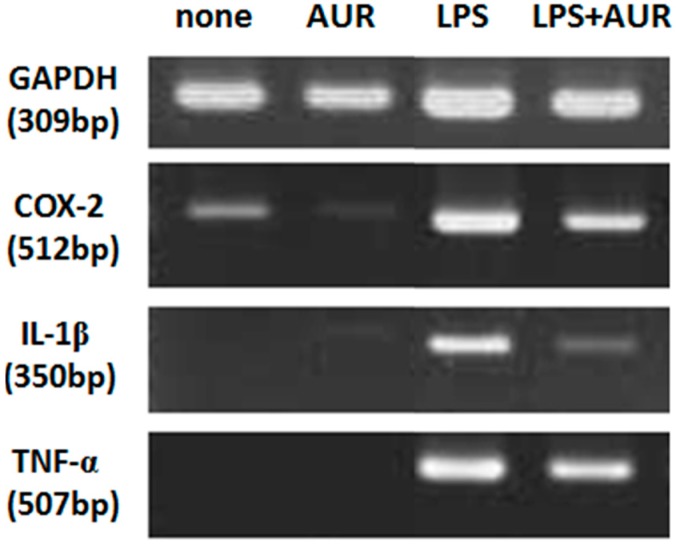
Effects of AUR on the expression of COX-2, IL-1β, and TNF-α mRNAs in cultured astrocytes. Cells were not treated or were treated with AUR (25 μM) alone for 30 min, with LPS (10 ng/mL) alone for 4 h, or with AUR (25 μM) for 30 min and then with LPS (10 ng/mL) for 4 h. Representative densitometric patterns of COX-2, IL-1β, TNF-α and GAPDH mRNAs bands were shown. Similar results were obtained from at least three independent experiments.

LPS has been shown to induce the production of inflammatory cytokines in macrophages [18]. Therefore, we investigated whether the AUR treatment effectively suppressed the LPS-induced expression of IL-1β mRNA and TNF-α mRNA in cultured astrocytes. Figure 4 revealed that no signals were detected for IL-1β mRNA or TNF-α mRNA in non-treated cells (lane of non-treated) or AUR-treated cells (lane of AUR). LPS markedly enhanced (lane of LPS), whereas AUR suppressed these increases. These results suggest that AUR exerts anti-inflammatory effects in cultured astrocytes. Inflammatory cytokines induce neuronal cell death following ischemia; therefore, the suppression of cytokine production in astrocytes by AUR was associated with neuronal cell protection in the hippocampal CA1 region.

This is the first study to demonstrate that AUR has anti-inflammatory effects on astrocytes *in vitro*. In activated RAW264 macrophages, AUR was recently shown to suppress inflammatory responses in peripheral tissues by inhibiting the activation of p38 mitogen-activated protein kinase [5]. We are now investigating the mechanisms responsible for the anti-inflammatory effects of AUR using cultured astrocytes.

### 2.2. Determination of AUR in the Mouse Brain after Its i.p. Administration

**Figure 5:**
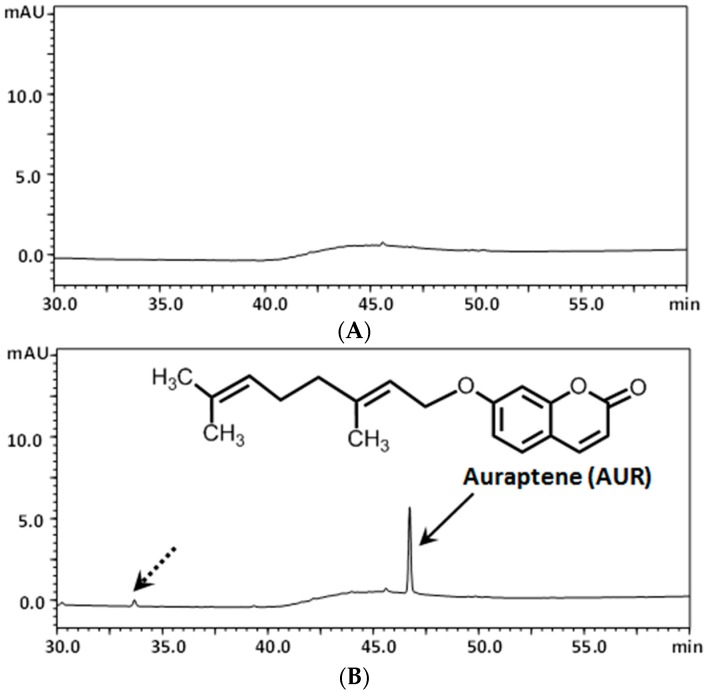
HPLC chromatograms of vehicle-treated brains (**A**); and brains from mice treated *i.p.* with AUR (**B**).

In order to confirm that AUR has the ability to pass through the BBB, mice were intraperitoneally (*i.p.*) administered AUR once (50 mg/kg). Figure 5A shows the typical chromatograms of non-treated brain samples, revealing the absence of interfering peaks. Figure 5B shows the typical chromatograms of AUR-spiked brain samples, revealing a sharp peak in AUR (solid arrow) and another small peak considered to be a metabolite of AUR (dashed arrow). This is the first study to demonstrate the permeability of AUR through the BBB.

**Figure 6:**
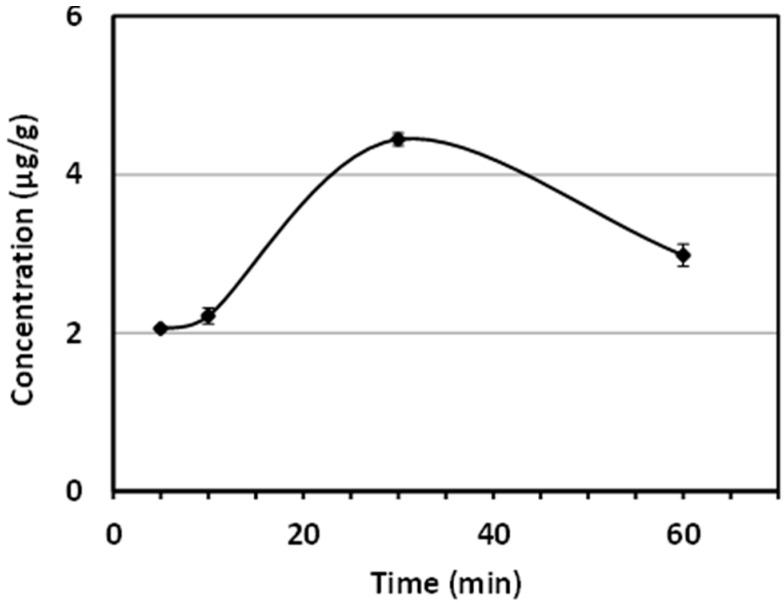
Time course of brain AUR concentration profiles following its *i.p.* administration (*n* = 3 at each time point).

The calibration curve for the standard of AUR was linear over the range of 0.01–0.0001 mg/mL. The correlation coefficient (*r*) was >0.999, as determined by a least square analysis, suggesting good linearity between the peak area ratio and AUR concentrations. The limit of quantification (LOQ) for AUR was *ca.* 0.02 μg/mL. Brain samples were analyzed using the HPLC conditions described above 5, 10, 30, and 60 min after the *i.p.* injection of AUR. Figure 6 shows that the brain tissue level of AUR gradually increased at 5 and 10 min, with the highest concentration (4.44 μg/g) being detected at 30 min. Figure 6 also shows that AUR was even detected after 60 min.

Small strokes and local chronic ischemia are frequently reported in the brains of elderly individuals [19]. Inflammation has been suggested to play a critical role in ischemic damage [20,21]. The accumulation of evidence has confirmed that inflammation is the key contributor to the pathophysiological process of not only ischemic strokes, but also various intracerebral diseases such as age-related neurological disorders, Alzheimer’s disease, multiple sclerosis, and brain tumors [19,22]. These findings suggest that the prevention of inflammatory reactions in the brain alleviates cognitive dysfunctions and other neurodegenerative processes in the elderly. The protective effects of AUR may offer a promising means to prevent chronic inflammation in the brain. Furthermore, most neurodegenerative diseases are generally progressive diseases with a late onset and of a long duration; therefore, the preemptive intake of AUR may prevent these diseases.

## 3. Experimental Section

### 3.1. Animal Preparation

Nine-week-old male C57BL/6 mice from Japan SLC (Hamamatsu, Japan) were used. Mice were kept under standard laboratory conditions of 23 ± 1 °C on a 12-h light/12-h dark cycle (lights on 8:00~20:00) with free access to food and water. All animal care and experimental protocols were approved by the Guidelines for Animal Experimentation and the Animal Care and Use Committee of Matsuyama University.

### 3.2. Brain Sample Preparation for HPLC/UV

AUR (LKT Laboratories, St. Paul, MN, USA) was dissolved in 10% dimethyl sulfoxide (DMSO)/saline solution, and *i.p.* administered (50 mg/kg) to mice (*n* = 3 at each time point). Mice were perfused with ice-cold phosphate-buffered saline (PBS) through the left ventricle 5, 10, 30, and 60 min after the administration of AUR, and then quickly sacrificed to excise the brains. These brain samples were stored at −80 °C before being used. Regarding sample preparation for the HPLC analysis, approximately 250 mg of each brain was sonicated with an equal weight of H_2_O for 30 s by Sonicator 3000 (Misonix Inc., Farmingdale, NY, USA). The homogenates (100 μL) were added to 150 μL of methanol, and then vortexed for 5 min. Supernatants were recovered after centrifugation at 14,000× *g* at 4 °C for 10 min.

### 3.3. Assessment of AUR via HPLC/UV

A stock solution of AUR was prepared by dissolving in methanol. The stock solution was diluted to 0.01–0.0001 mg/mL for validation measurements. A HPLC analysis was performed using a Shimadzu Prominence system (Shimadzu, Kyoto, Japan). Reversed-phase (RP) HPLC conditions were as follows: column, L-column ODS (5 μm, 150 × 2.1 mm i.d.) (Chemicals Evaluation and Research Institute, Tokyo, Japan); mobile phase, solvent A was 5% acetic acid and solvent B was acetonitrile (0–30 min, 0–50% B in A; 30–35 min, 50%–85% B in A; 35–40 min, 85%–85% B in A); injection volume, 2 μL; column temperature, 40 °C; flow rate, 0.3 mL/min; detection, 320 nm.

### 3.4. Procedures for Ischemic Surgery

Previously described procedures for 2VO [17] were adopted for ischemic surgery. Throughout surgery, body temperature was maintained at 37 ± 0.5 °C with a heating pad, and brain surface temperature was monitored with a tympanic membrane probe into the ear and maintained at 36.5 ± 0.2 °C. After surgery, all mice were placed in a recovery cage under a heat lamp and had free access to drinking water.

AUR was dissolved in DMSO/polyethylene glycol (PEG) (1:1) solutions. Animals were randomly divided into four groups. Mice in the sham-control group (Sham; *n* = 9) and 2VO-control group (2VO; *n* = 10) were *s.c.* administered vehicle with an implanted osmotic pump (Alzet 1007D; DURECT Corporation, Cupertino, CA, USA) operated at a speed of 0.5 μL/h. Mice in the 2VO surgery and AUR (10 mg/kg/day)-treatment group (2VO + AUR10; *n* = 8) and 2VO surgery and AUR (25 mg/kg/day)-treatment group (2VO + AUR 25; *n* = 11) were *s.c.* administered AUR solution with an osmotic pump operated at the same speed. Osmotic pump implantation and the infusion of samples were initiated five days before ischemic surgery, and the treatment was continued for another three days.

### 3.5. Immunohistochemistry

Mice were killed as previously described in detail [8]. Whole brains were separated, frozen, and then sectioned (thickness of 30 μm). Sagittal sections were stained with the desired antibodies. In order to stain microglia, we used a rabbit polyclonal antibody against IBA1 (1:1000; Wako, Osaka, Japan) as the primary antibody, followed by an EnVision-plus system-HRP-labeled polymer (anti rabbit; Dako, Glostrup, Denmark) as the secondary antibody. Immunoreactivity was visualized using a DAB substrate (SK-4100; Vector Laboratories, Burlingame, CA, USA). Hippocampal images were obtained with a CX21 microscope (Olympus, Tokyo, Japan).

In order to stain for COX-2, we used a goat monoclonal antibody against COX-2 (1:50; Santa Cruz Biochemistry, Santa Cruz, CA, USA) as the primary antibody, with Alexa Fluor 488-labeled donkey anti-goat IgG (H + L; 1:300; Invitrogen, Carlsbad, CA, USA) as the secondary antibody. Mouse GFAP (1:200; Sigma-Aldrich, St. Louis, MO, USA) was used to stain astrocytes, with Alexa Fluor 568-labeled goat anti-mouse IgG (H + L, 1:300; Invitrogen) as the secondary antibody. A mounting medium was used (Vectashield; Vector Laboratories). Images of the hippocampus were captured with a confocal fluorescence microscopy system (LSM510; Zeiss, Oberkochen, Germany).

### 3.6. Cell Culture

Astrocytes were cultured from the cerebral cortex of one- to three-day-old ICR mice (Japan SLC) and maintained in Dulbecco’s modified Eagle’s medium (DMEM; Sigma-Aldrich) containing 10% fetal calf serum (FCS; ICN Biochemicals, Aurora, OH, USA) as described previously [23]. Approximately 10 days later, cells were sub-cultured on 6-well plates and maintained in a humidified atmosphere (5% CO_2_) at 37 °C. After reaching confluence, these cells were cultured for approximately one week in FCS-free DMEM containing 0.5% bovine serum albumin (BSA). Astrocytes were then exposed to AUR and/or LPS. AUR was dissolved in DMSO to prepare the stock solution (100 mM) as previously described [24]. LPS was dissolved in saline.

### 3.7. RT-PCR Procedures

Total RNA from hippocampal tissue and cultured astrocytes was prepared as previously described [25] and transcribed into cDNA using the SMART PCR cDNA Synthesis Kit (Clontech, Palo Alto, CA, USA). The synthesized cDNA was amplified by PCR using each primer pair. The following primer pairs were used: 5’-cttgggctgtccagatgagagcat-3’ and 5’-gaagacacgggttccatggtgaag-3’ for COX-2; 5’-gccgtcttcccctccatcgt-3’ and 5’-cccgtctccggagtccatca-3’ for β-actin; 5’-cttgggctgtccagatgagagcat-3’ and 5’-gaagacacgggttccatggtgaag-3’ for IL-β; 5’-atgagcacagaaagcatgat-3’ and 5’-tgactttctcctggtatga for TNF-α; and 5’-cggagtcaacggatttggtcgtat-3’ and 5’-agccttctccatggtggtgaagac-3’ for GAPDH. Regarding brain tissues, the numbers of PCR cycles and specific annealing temperatures were 35 cycles and 56 °C for COX-2 and 36 cycles and 60 °C for β-actin. The numbers of PCR cycles and specific annealing temperatures for cultured astrocytes were 36 cycles and 56 °C for COX-2, 34 cycles and 61 °C for IL-β, 35 cycles and 50 °C for TNF-α, and 29 cycles and 55 °C for GAPDH. Reaction products were electrophoresed on 2% agarose gels containing ethidium bromide. The intensity of staining was measured using the LAS-3000 imaging system (Fujifilm, Tokyo, Japan).

### 3.8. Statistical Analysis

Data were expressed as means ± SEM. The significance of differences was analyzed using a one-factor ANOVA followed by Bonferroni’s Multiple Comparison Test (Prism 5; GraphPad Software, La Jolla, CA, USA). The criterion for significance was *p* < 0.05 in all statistical evaluations.

## 4. Conclusions

We recently reported that peripherally administered AUR, a citrus coumarin, may have anti-inflammatory effects in the mouse brain. However, the penetration of AUR into the brain has not yet been examined. The present study evaluated the amounts of AUR in the brains of mice following its *i.p.* administration, indicating its ability to penetrate the brain. The present study also showed *in vivo* that AUR, as a *s.c.* pretreatment (25 mg/kg/day) for five days before and for another three days after ischemic surgery, suppressed microglial activation and COX-2 expression in the hippocampal region. Using cultured astrocytes, we showed *in vitro* that AUR had suppressive effects on the mRNA expression of the inflammatory cytokines IL-1β and TNF-α as well as COX-2. Taken together, these results support our previous suggestion that AUR has the ability to directly exert anti-inflammatory effects in the brain.
